# Atrial Myxoma: An Unusual Etiology of Ischemic Stroke in an Adult Patient

**DOI:** 10.7759/cureus.40599

**Published:** 2023-06-18

**Authors:** Haleema Sadia, Adetola F Oshikoya, Priyanka Sachdev, Oluwatobiloba F Fasoranti-Sowemimo, Saima H Shawl, Kapil Kumar, Alaa S Mohamed, Muhammad Haseeb, Hira Nasir

**Affiliations:** 1 Internal Medicine, Khyber Teaching Hospital Peshawar, Peshawar, PAK; 2 Internal Medicine, Near East University, Nicosia, CYP; 3 Internal Medicine, General Hospital Odan, Lagos, NGA; 4 Internal Medicine, Liaquat University of Medical and Health Sciences, Jamshoro, PAK; 5 Medicine and Surgery, Obafemi Awolowo University, Ile-Ife, NGA; 6 Internal Medicine, Midwest Sleep and Wellness Clinic, Chicago, USA; 7 Medicine, Chattogram Medical College Hospital (CMCH), Chittagong, BGD; 8 Medicine and Surgery, Liaquat National Hospital and Medical College, Karachi, PAK; 9 Neurology, Augusta University, Augusta, USA; 10 Medicine, Allama Iqbal Medical College, Lahore, PAK; 11 Internal Medicine, Mount Sinai Hospital, Brooklyn, USA; 12 Internal Medicine, Mayo Hospital, Lahore, PAK

**Keywords:** echocardiogram, ischemic stroke, cardiovascular, neuroembolism, cardiac myxoma

## Abstract

Atrial myxoma is the most frequent primary cardiac tumor; however, it is a rare, substantial cause of cardiogenic emboli causing a stroke, especially in young adults. A cardiac myxoma has no specific clinical presentation, ranging from constitutional symptoms to non-cardiac symptoms and emboli, which leads to a diagnostic challenge in the clinical process. We report a case of a left atrial myxoma in an adult female who presented with sudden onset of right-sided weakness, headache, and numbness. Imaging confirmed cardiogenic emboli from the cardiac myxoma, which was reflected in an ischemic stroke.

## Introduction

An estimated 14-30% of ischemic strokes are caused by cardioembolism, which has the tendency to occur in both early and late stages [1]. Cardiac causes responsible for cardioembolic stroke through cerebral emboli include valvular heart disease, atrial fibrillation, infective endocarditis, myocardial infarction, and atrial cardiac myxoma [2]. The most common one is atrial fibrillation, which accounts for almost 45% of cardioembolic strokes [3]. Atrial myxoma is the most frequent primary cardiac tumor; however, it is a rare, substantial cause of cardiogenic emboli causing a stroke in individuals, especially in young adults. Cardiac myxoma is a benign and slow proliferating neoplasm with a low incidence, approximately 0.5-1% cases per million per year [2,3]. Cardiac myxoma has no specific clinical presentation, ranging from constitutional symptoms to non-cardiac symptoms and emboli, which leads to a diagnostic challenge in the clinical process [4]. We report a case of an ischemic stroke in a young patient caused by cardiogenic emboli from atrial myxoma.

## Case presentation

A 44-year-old female with a history of primary hyperthyroidism was brought to the emergency department with sudden onset of right-sided weakness for the last eight hours, followed by intermittent headache, numbness, and heaviness in the right limbs. She was compliant with her carbimazole and reported no history of smoking, substance, or alcohol abuse. She referred to an unintentional weight loss of 20 pounds over the last four months. She denied any family history of similar disease and had no previous history of seizure, syncope, headache, or incontinence.

**Figure 1:**
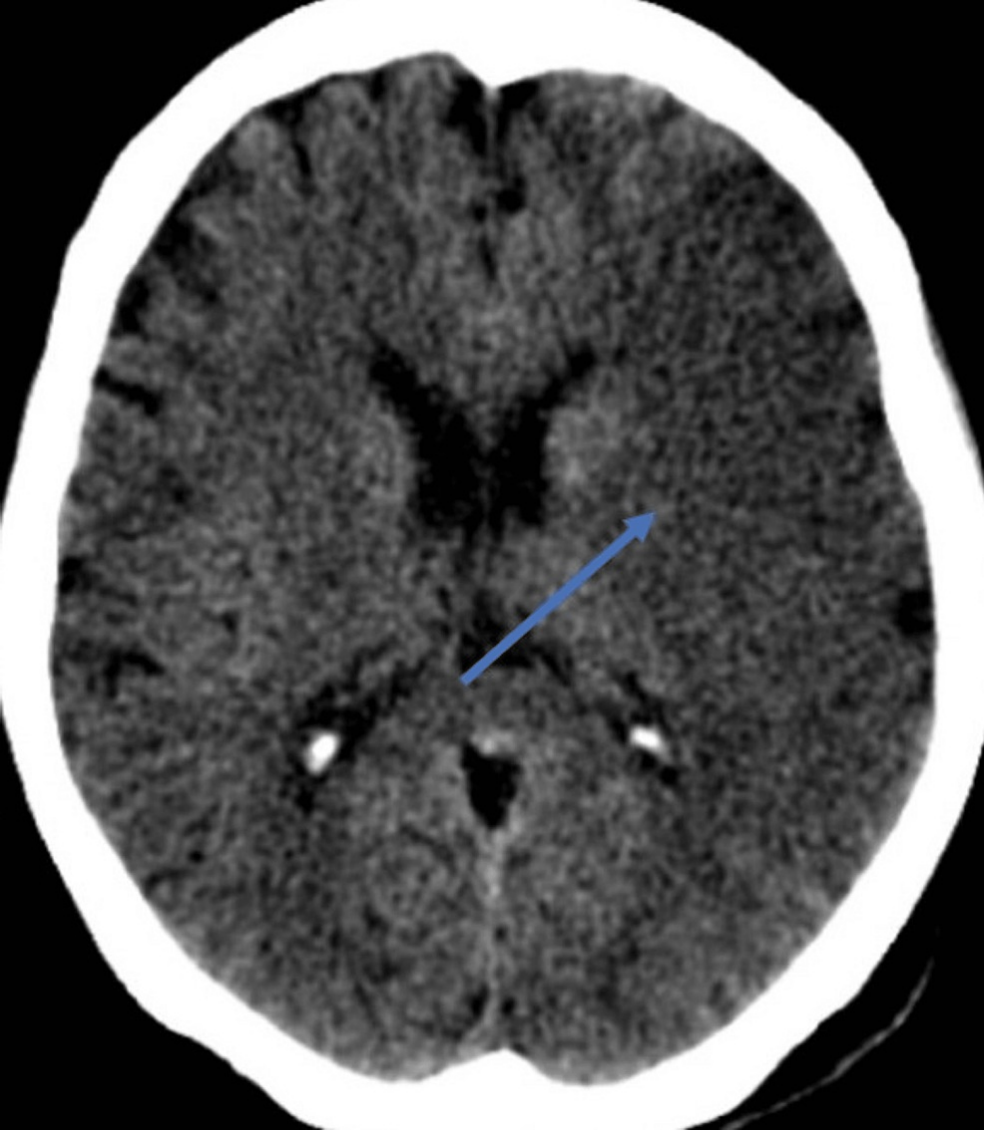
Brain CT showing hyperdense areas in the left cerebral hemisphere CT: computed tomography

On examination, she was hemodynamically stable except for high blood pressure (170/105 mmHg) and well-oriented to time, place, and person. She had an intact cranial nerve examination and had no signs of meningeal irritation. There was right-sided hemiplegia and extensor right-sided plantar reflex, power of 1/5 in the upper and 2/5 in the lower limbs, as well as hypoesthesia in the upper limb. Her reflexes were normal in all extremities. Cardiovascular and respiratory examinations, including thyroid, were unremarkable. The electrocardiogram revealed sinus rhythm and the chest X-ray was normal. Initial blood investigations, including thyroid function tests, were nonsignificant. A provisional diagnosis of stroke was made, and she underwent emergent non-contrast brain computed tomography (CT), which revealed an ischemic stroke of the left cerebral hemisphere (Figure 1).

**Figure 2:**
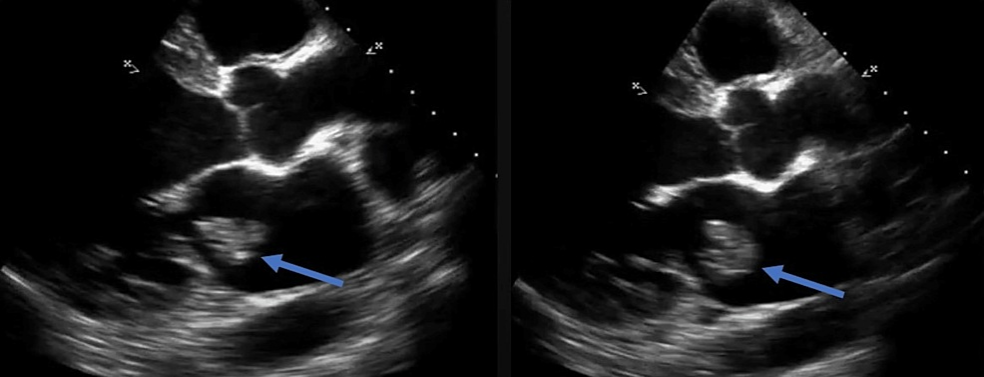
Transthoracic echocardiography demonstrating intracardiac mass in the left atrium, suggestive of atrial myxoma (blue arrows)

On further assessment of the stroke, she underwent transthoracic echocardiography (TTE), which revealed normal systolic and diastolic function of the heart with an ejection fraction of 55%. A mobile and pedunculated mass was noted, measuring 3.65 x 2.5 cm in the left atrium attached to the atrial septum, which was intruding on the mitral valve orifice (Figure 2). A probable diagnosis of atrial myxoma was made.

**Figure 3:**
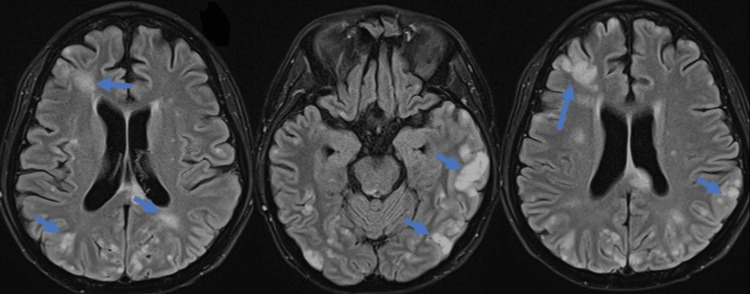
MRI brain demonstrating multiple small foci of brain infarcts in all the territory of the major vasculature, particularly in the cortical and peripheral distribution (blue arrows) MRI: magnetic resonance imaging

She underwent non-contrast brain magnetic resonance imaging, which revealed an acute infarction in the territory of the middle and posterior cerebral artery involving the left cerebral hemisphere (Figure 3). A consultation was ordered with a cardiothoracic surgeon based on the patient's clinical presentation and her imaging finding. Coronary angiography was advised before the planned surgical removal of the intracardiac mass. Her coronary angiography was normal except for minimal stenosis in the right coronary artery.

**Figure 4:**
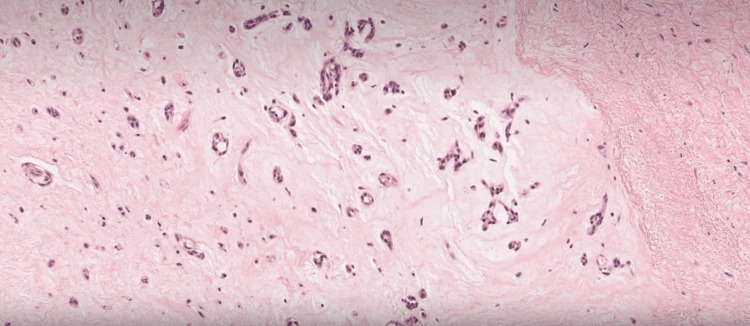
Histopathology demonstrating myxoma cells surrounded by spindle cells, suggesting atrial myxoma

Initially, she was managed with unfractionated heparin and was commenced on lisinopril and atorvastatin based on her clinical status. She underwent cardiac surgery, and the atrial myxoma was removed without complications. Histopathology of the lesion confirmed cardiac myxoma, and she was discharged on aspirin and symptomatic management. On her three-month follow-up, her neurological manifestations improved (Figure 4).

## Discussion

Atrial myxoma is a primary cardiac tumor arising from the endocardium, typically found in the left atrium [1]. It is the most benign cardiac tumor, accounting for approximately half of all cardiac tumors. Being the most common cardiac tumor, atrial myxoma is observed more frequently in young patients presenting with cardioembolic stroke, with an incidence of 0.004%, compared to older patients with similar presentations, with an incidence of 0.001% [5]. Atrial myxoma occurs more frequently in women, with a female-to-male ratio of approximately 2:1; however, tumors can affect individuals of all ages, with peak incidence observed in the fourth to six decades of life [4]. Atrial myxoma is characterized by a gelatinous or myxoid consistency due to a combination of stellate cells, connective tissue, and mucopolysaccharides [1]. The tumor commonly arises from a pedunculated stalk attached to the atrial septum. The mobility of the tumor allows it to protrude into the left ventricle during diastole and retract into the left atrium during systole. Although the exact etiology of atrial myxomas remains unknown, genetic and sporadic mutations have also been proposed as potential contributors [5,6].

Atrial myxoma is one of the important causes of cardioembolic stroke in young populations. In a case study of 113 patients diagnosed with atrial myxoma and neurological manifestations, Singh et al. highlighted that most patients presented with ischemic stroke, followed by syncope, psychiatric manifestations, and seizures [7]. Lee et al. highlighted, in a research article of 59 patients diagnosed with cardiac myxoma, that 22% experienced a cardioembolic phenomenon, and only two patients developed stroke [8]. Moss et al. reported a case of cardiac myxoma in a middle-aged patient who presented with sudden left-sided paresis and paresthesia and was diagnosed with multifocal ischemic strokes [9]. Sohal et al. also underlined a case of ischemic stroke in a young patient caused by cardiogenic emboli from an atrial myxoma [10]. The underlying pathophysiological mechanism of cardiac myxoma as an etiology of ischemic stroke is the friable and gelatinous nature of the tumor component, which can dislodge from the tumor causing embolization to distant areas. Other mechanisms include hemodynamic alterations, activation of the coagulation cascade, and immunological response triggered by the tumor [8].

Atrial myxoma manifestations often constitute a diagnostic triad (Table 1) [6,7,11]. The clinical presentation can vary depending on the size, location, and number of emboli. Common symptoms include sudden onset focal neurological deficits such as paresis, dysarthria, and sensory or visual abnormalities [4]. The severity and duration of the manifestations depend on the degree of vessel occlusion and the promptness of the intervention. Atrial myxoma can also present with constitutional symptoms, which may mimic systemic inflammatory conditions, and the presence of such symptoms along with neurological deficits should raise the suspicion of cardiogenic emboli [12].

**Table 1:** Diagnostic triad in the initial presentation of atrial myxoma

Features	Clinical Manifestations	Frequency (%)
Embolic phenomenon	Cardiogenic emboli to any organ; however, 73% embolize to the central nervous system	10-45
Constitutional symptoms	Myalgia, arthralgia, fatigue, fever, weight loss, anorexia, clubbing, autoimmune disease features	34-90
Obstructive symptoms	Syncope, dyspnea, heart failure, sudden death	54-95

Diagnosing ischemic stroke induced by atrial myxoma involves a multidisciplinary approach, including clinical evaluation, neuroimaging, and cardiac investigations. Neuroimaging is crucial in confirming the diagnosis and evaluating the extent of cerebral involvement. TTE is the first-line imaging modality to assess cardiac structures and identify an atrial myxoma, which can visualize the tumor and its characteristics, including size, location, and mobility [13]. Managing an ischemic stroke induced by cardiogenic emboli involves anticoagulation and modification of risk factors to prevent further embolic events while awaiting definitive treatment. Surgical removal of tumors is the mainstay of treatment, including the prevention of new events. The prompt surgical intervention aims to prevent recurrent embolic events and minimize the risk of further neurological deterioration [14].

## Conclusions

Although rare, cardiac myxoma should be included in the differential diagnosis as an etiology of ischemic stroke in young patients who present with sudden-onset paresis, dysarthria, seizures, or paresthesia. A prompt diagnosis is essential in order to achieve an excellent prognosis and the timely management of an ischemic stroke, and managing the underlying condition is mandatory to prevent severe morbidity and mortality. Further data from the ongoing studies are required to focus on refining the imaging modalities, investigating the use of novel anticoagulants, and evaluating the role of minimally invasive surgical techniques.
